# Effects of Microenvironment and Dosing on Efficiency of Enhanced Cell Penetrating Peptide Nonviral Gene Delivery

**DOI:** 10.1021/acsomega.3c09306

**Published:** 2024-01-18

**Authors:** James E. Dixon, Vanessa Wellington, Alaa Elnima, Hoda M. Eltaher

**Affiliations:** † Regenerative Medicine and Cellular Therapies Division, The University of Nottingham Biodiscovery Institute (BDI), School of Pharmacy, University of Nottingham, Nottingham NG7 2RD, U.K.; ‡ NIHR Nottingham Biomedical Research Centre, University of Nottingham, Nottingham NG7 2RD, U.K.

## Abstract

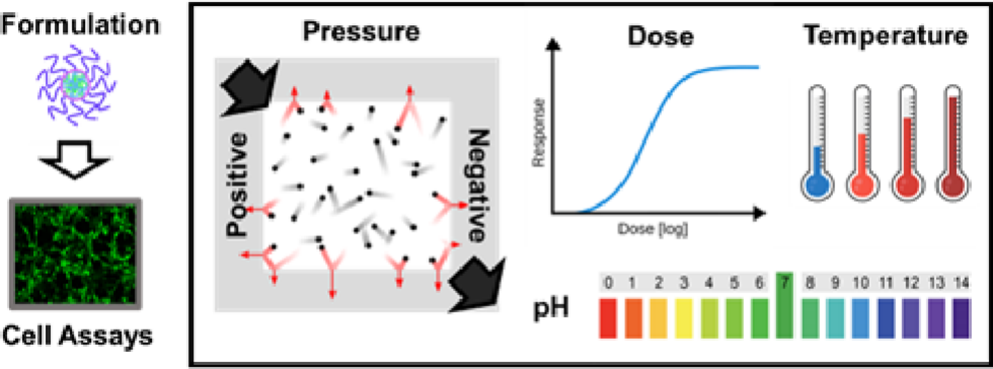

Transfection, defined as functional delivery of cell-internalized nucleic acids, is dependent on many factors linked to formulation, vector, cell type, and microenvironmental culture conditions. We previously developed a technology termed glycosaminoglycan (GAG)-binding enhanced transduction (GET) to efficiently deliver a variety of cargoes intracellularly, using GAG-binding peptides and cell penetrating peptides (CPPs) in the form of nanoparticles, using conventional cell culture. Herein, we demonstrate that the most simple GET transfection formulation (employing the FLR peptide) is relatively poor at transfecting cells at increasingly lower dosages. However, with an endosomally escaping version (FLR:FLH peptide formulations) we demonstrate more effective transfection of cells with lower quantities of plasmid (p)DNA *in vitro*. We assessed the ability of single and serial delivery of our formulations to readily transfect cells and determined that temperature, pH, and atmospheric pressure can significantly affect transfected cell number and expression levels. Cytocompatible temperatures that maintain high cell metabolism (20–37 °C) were the optimal for transfection. Interestingly, serial delivery can maintain and enhance expression without viability being compromised, and alkaline pH conditions can aid overall efficiencies. Positive atmospheric pressures can also improve the transgene expression levels generated by GET transfection on a single-cell level. Novel nanotechnologies and gene therapeutics such as GET could be transformative for future regenerative medicine strategies. It will be important to understand how such approaches can be optimized at the formulation and application levels in order to achieve efficacy that will be competitive with viral strategies.

## 1 Background

Macromolecular drugs, such as peptides and nucleic acids, are highly specific, potent agents that have shown great promise as novel therapeutics in the treatment of many diseases.^1^ These could offer many advantages compared with small molecule drugs with high potency, low nonspecific activity, and toxicity;^2^ however, their clinical use has been inhibited due to poor overall function when delivered. Specifically, nucleic acids like DNA or RNA have short *in vivo* circulation half-life and biodistribution, and are rapidly destroyed through physical and chemical degradation. The lack of an efficient, safe, specific, and universal delivery platform without using viral systems prevents their impact on medicine. In addition, further issues such as reticuloendothelial system-mediated clearance, vector immunogenicity, poor solubility, and failure to penetrate both tissue and cellular membranes effectively further reduce their therapeutic efficacy.^3^ For nonviral gene delivery to achieve a high therapeutic efficacy, novel delivery platforms to mitigate these defects are vital.

Various methodologies have been developed to deliver therapeutic proteins and nucleic acids intracellularly using nanotechnology approaches.^4−9^ Cell penetrating peptides (CPPs), often known as protein translocation domains or Trojan peptides, are successful in delivering variable cargoes^10^ where they can be linked to therapeutics^11^ and trigger endocytosis-mediated uptake.^4^ Examples include the cationic amphipathic peptide, RALA^12^ or the efficient molecular cargoes transporter, Penetratin.^13^ Even though CPPs significantly increase uptake, efficacy often requires vast extracellular excess (at micromolar scales) to drive significant endocytosis. We have described the glycosaminoglycan (GAG)-binding enhanced transduction (GET)^14^ system that exploits enhanced membrane-docking peptides that bind heparan sulfate GAGs, conjugated with CPPs to generate nanoformulations. We have demonstrated that functional quantities of many cargos can be delivered to cells. Furthermore, the GET system can be employed in conventional media,^14−17^ scaffolds,^18^ biomaterials,^15,19^ and encapsulated within hydrogels.^20,21^ GET nanoparticles (formed with complexed nucleic acids) have been shown to deliver plasmid (p)DNA and mRNA having high transfection efficiency *in vitro* or *in vivo*.^22−25^ We have shown that by generating PEGylated versions, the system can achieve effective lung gene expression^22^ by possessing reduced extracellular trapping, with enhanced diffusion. This is achieved by shielding the particle’s cationic properties. Furthermore, endosomally escaping formulations (incorporating the peptide FLH; FGF2B-LK15–10H) have been engineered to promote functions that have most impact in gene delivery to difficult-to-transfect target cells.^25^

Here, in this study, we tested the effectiveness of suboptimal doses of GET formulations (FLR and FLR:FLH) to mediate effective gene delivery. We discovered that endosomal-escape-enhanced versions had significantly increased transfection at lower dosages. We tested environmental conditions, such as temperature during delivery, pH, culture atmosphere, and pressure, in order to understand the importance of these conditions in efficient transfection. We also demonstrated that serial dosing is possible, and can augment and retain high levels of transgene expression. Understanding the optimal environment will allow nonviral approaches to be robustly employed for gene therapies and realize the potential of new genetic technologies and editing strategies.

## 2 Materials and Methods

### 2.1 Materials

All materials were purchased from Sigma-Aldrich (UK) unless stated. Dulbecco’s phosphate-buffered saline (DPBS) was provided by ThermoFisher. Pipework and connectors were obtained from Silex Silicones Ltd. (UK).

### 2.2 Cell Culture

NIH3t3 fibroblast cells (ATCC-CRL-1658) were cultured in Dulbecco’s modified Eagles media (DMEM; Sigma), supplemented with 10% (v/v) fetal bovine serum (FBS, Sigma), 4.5 g/L d-glucose, 2 mM l-glutamine, and 100 units/mL penicillin and 100 units/mL streptomycin (Invitrogen). This media was defined as growth media (GM), the same media without FBS was defined as serum-free media (SFM) and cells were cultured at 37 °C and humidified 5% CO_2_ as described previously.^14^ For CO_2_ independent media (CIM; Invitrogen, cat. no. 18045088) and pH range media experiments, cells were plated in GM and switched to CIM (containing 10% FBS and 100 units/mL penicillin and 100 units/mL streptomycin or pH-adjusted GM) before treatment. pH was adjusted by the addition of 1 M HCl or NaOH and the media was filtered with a 0.4 μm syringe-filter.

### 2.3 Cell Metabolism and Viability

Cell viability from monolayers were assayed for cell metabolism using PrestoBlue (ThermoFisher, cat no: A13262) as described previously.^26,27^ We employed 50 μL and 500 μL volumes for assays of 96-well plates and 12-well plates, respectively. Time of incubation was varied with appropriate controls to allow significant color changes before fluorometry in black 96-well plates (50 μL/sample). Cell suspensions were assayed for viability using trypan-blue exclusion and hemocytometer assessment. LIVE/DEAD (ThermoFisher, cat no. L3224) was used following manufacturers’ instructions with modifications detailed previously.^28^

### 2.4 GET Peptides and pDNA Preparation

FLR (TYRSRKYTSWYVALKRKLLKLLLKLLLKLLKRRRRRRRR) and FLH (TYRSRKYTSWYVALKRKLLKLLLKLLLKLLKHHHHHHHHHH) peptides were synthesized as previously described.^17,22^ For luciferase assays, reporter plasmid (pDNA) expressing *gaussia luciferase (gluc)* was acquired from New England Biolabs (pCMV-gluc2 termed pGluc).^23^ For fluorescent reporter assays, *enhanced green fluorescent protein (eGFP)* expressing pDNA was acquired from Takada, Japan (pEGFP-C1 termed peGFP). Both plasmids were driven by an enhanced cytomegalovirus (CMV) promoter. The plasmids were transformed in DH5α competent *E. coli* cells and purified by endofree Maxi-prep kits (Qiagen, UK) as previously.^22^

### 2.5 GET Nanoparticle Complexation and Transfection

Our conventional GET nanoparticle complexation methodology was modified and scaled to the volumes required.^22^ Typically for 96-well transfections, we used high cell densities (2.5 × 10^4^ NIH3t3 cells) and delivered 0.125 μg of plasmid (p)DNA (in 6.25 μL of SFM) complexed with 0.1 μL of FLR (for FLR) with an additional 0.125 μL of FLH (1 mM) (in a total volume of 6.25 μL with SFM) (for FLR:FLH) creating a 12.5 μL transfection volume. These were then combined, mixed, complexed for 15 min at room temperature, and then added to samples (containing 50 μL media). This cell-exposed concentration was defined as 1× (2 μg/mL). The maximum pDNA concentration used for complexation was 4 μg pDNA in 12.5 μL to enable faithful GET nanoparticle generation. For higher dosage experiments, larger complexation volumes were employed.

### 2.6 Pressure

Negative pressure (NP) was achieved by placing samples in a vacuum oven (with NP adjusted using a vacuum pump) (37 °C and humidified with 5% CO_2_) after the addition of transfection complexes. Positive pressure (PP) was achieved using a prewarmed paint resin tank (with PP adjusted using compressed air). Transfection was added and the samples were placed in the tank, which were pressurized with an air compressor and placed at 37 °C (humidified with a prewarmed water tray).

### 2.7 Luciferase Reporter Assays

Secreted luciferase reporter levels were measured 24 h post-transfection by plate-reader luminometer (TECAN Infinity) and compared with controls (as previously described).^22,23^

### 2.8 Fluorescence Microscopy and Flow Cytometry

Enhanced GFP fluorescence in cells was assessed by fluorescence microscopy and flow cytometry. Transfected cells as monolayers were washed twice with PBS and imaged by fluorescent microscopy (Leica DM IRB) using a blue laser for GFP. For flow cytometry, monolayer cultured cells were trypsinized with trypsin/EDTA (0.25% (w/v) trypsin/2 mM EDTA) and fixed with 4% (w/v) paraformaldehyde (PFA). GFP reporter expression was quantified using a Beckman Astrios Cell Sorter and 590 nm laser (20,000 cells minimum, gated on untreated cells by forward/side scatter). Mean fluorescence intensity was used for statistical analysis. Scatter plots and histogram graphs were produced by using Weasel flow cytometry analysis software.

### 2.9 Statistical Analysis

Statistical analysis and graphs were generated using the GraphPad Prism software package. Unpaired *t* test and one-way ANOVA were used to determine significant variances between two groups or more. Two-way ANOVA was used for grouped data. One-way and two-way ANOVA were followed by Tukey’s test to determine significance between each mean in multiple comparison. The data were represented as mean ± SD. Variances between means were considered to be statistically significant with *p-*values: 0.05 (*) and 0.01 (**). Experimental numbers were a minimum of three biological replicates in every experiment.

## 3 Results

### 3.1 Low Dose Transfection Success with FLR:FLH GET Formulations

**Figure 1:**
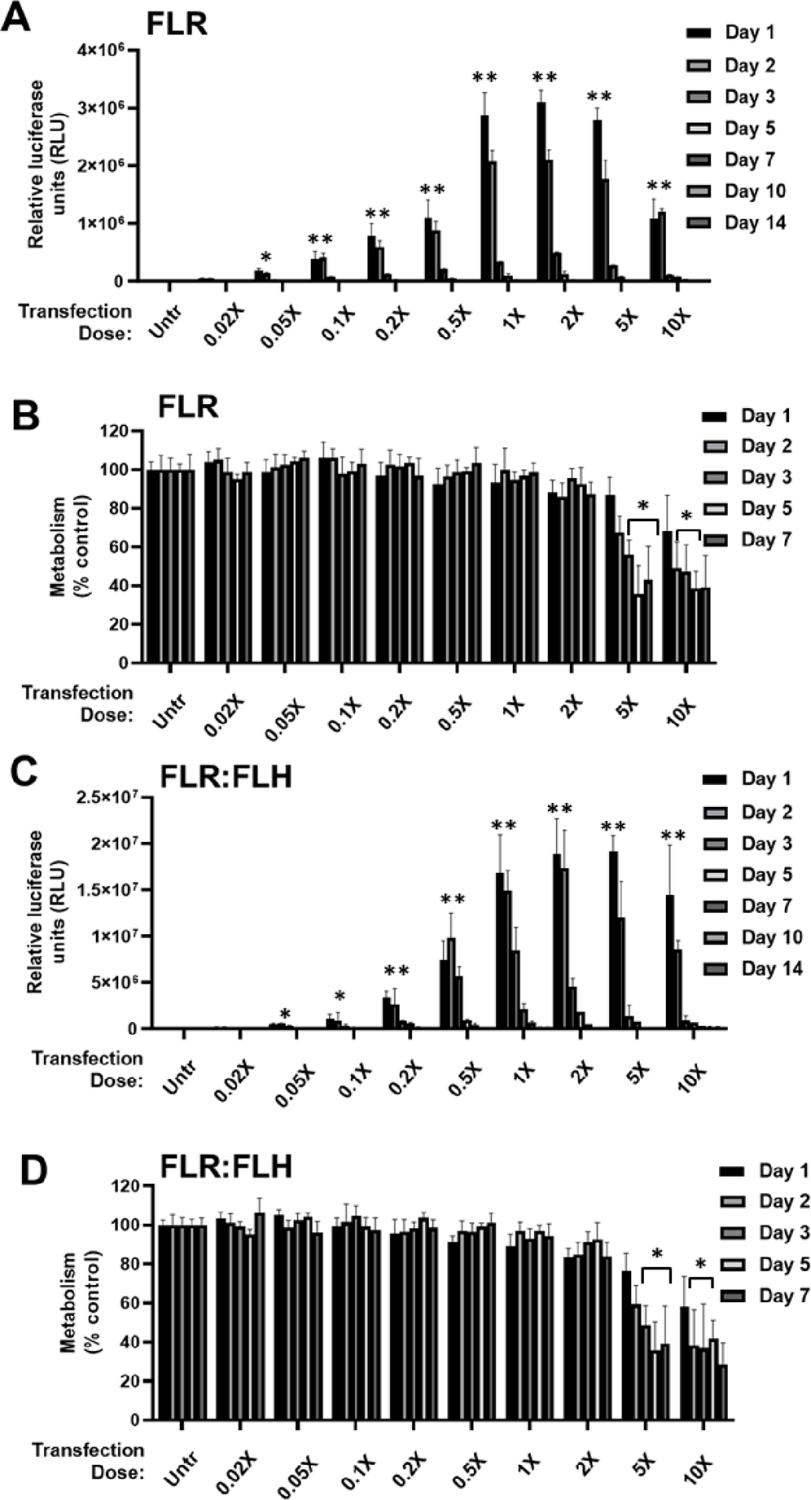
Optimizing FLR and FLR:FLH pDNA dose for transfection of NIH3t3 monolayers. (A) Luciferase assay of pGluc pDNA transfection using FLR at days 1, 2, 3, 5, 7, 10, 14 post-transfection in relative luciferase units (RLU). 1× dose was 2 μg/mL. Most conditions had lost significant reporter expression by day 14. (B) Metabolic activity (PrestoBlue) of NIH3t3 cell monolayers transfected as in (A) to day 7 post-transfection. (C) and (D) are experiments repeated for FLR:FLH. Data were normalized to untransfected (Untr) as 100% for each day (*N* = 6, bars are SD; **p < 0.01, *p < 0.05).

We first focused on the optimal formulation of GET. Initially, we assessed the transfection efficiency of NIH3t3 cells with a variant of GET peptides that had enhanced endosomal escape (FLR:FLH), compared to our conventional formulations (FLR only) (Figure 1).^25^ We transfected cells under these conditions using GET nanoparticles to deliver *gaussia luciferase* reporter pDNA (pGluc). Conventional transfections employ doses of 0.125 μg/well pDNA delivery using FLR (defined as a 1× dose for 2.5 × 10^4^ cell in 96-well plate format; 5 μg pDNA/1 × 10^6^ cells). We assessed FLR compared to FLR:FLH formulations over the range of a single 0–10× dose for 14 days. FLR:FLH formulations generated significantly higher transfection levels at the lowest dosages, whereas FLR was comparable at 1–5× doses. For FLR:FLH, the lowest dose to exhibit significant transfection over background was 0.02× (2.5 ng dose), which proportionally increased to plateau at the 2× (0.25 μg) dose (Figure 1A,C). Higher concentrations remained transfection competent but inhibited metabolism to ∼58.1% levels with 10× doses at day 1 post-transfection (Figure 1B,D). We selected FLR:FLH-based formulations (1× dose) for future studies, as this was more effective in the model cell line and transfection was detectable by luciferase with very low dose transfections.

### 3.2 The Effect of Atmosphere, Temperature, and pH on Cell Transfection and Viability

In order to apply a variety of microenvironmental conditions during transfection, we initially assessed how temperature, pressure, and pH could be tightly controlled. There are significant technical difficulties in precisely maintaining atmospheric and temperature conditions over hours to days using conventional culture. We therefore initially assessed the effect of moving cell incubations from conventional culture incubators (with 5% CO_2_ at 37 °C) to atmospheric (0.04%) or 5% CO_2_ at room-temperature (oven or incubator set at 25 °C, respectively) and atmospheric body-temperature (37 °C) conditions (Figure S1). We assessed metabolic activity with resazurin-based alamar/prestoblue assays as previously.^29^ Irrelevant of CO_2_ that had no effect on metabolism over 24 h incubation, 20–25 °C conditions inhibited metabolic activity (75.9% of control) (Figure S1A), with a nonsignificant increase (2.3%) in dead cells by live/dead analyses (Figure S1B).

**Figure 2:**
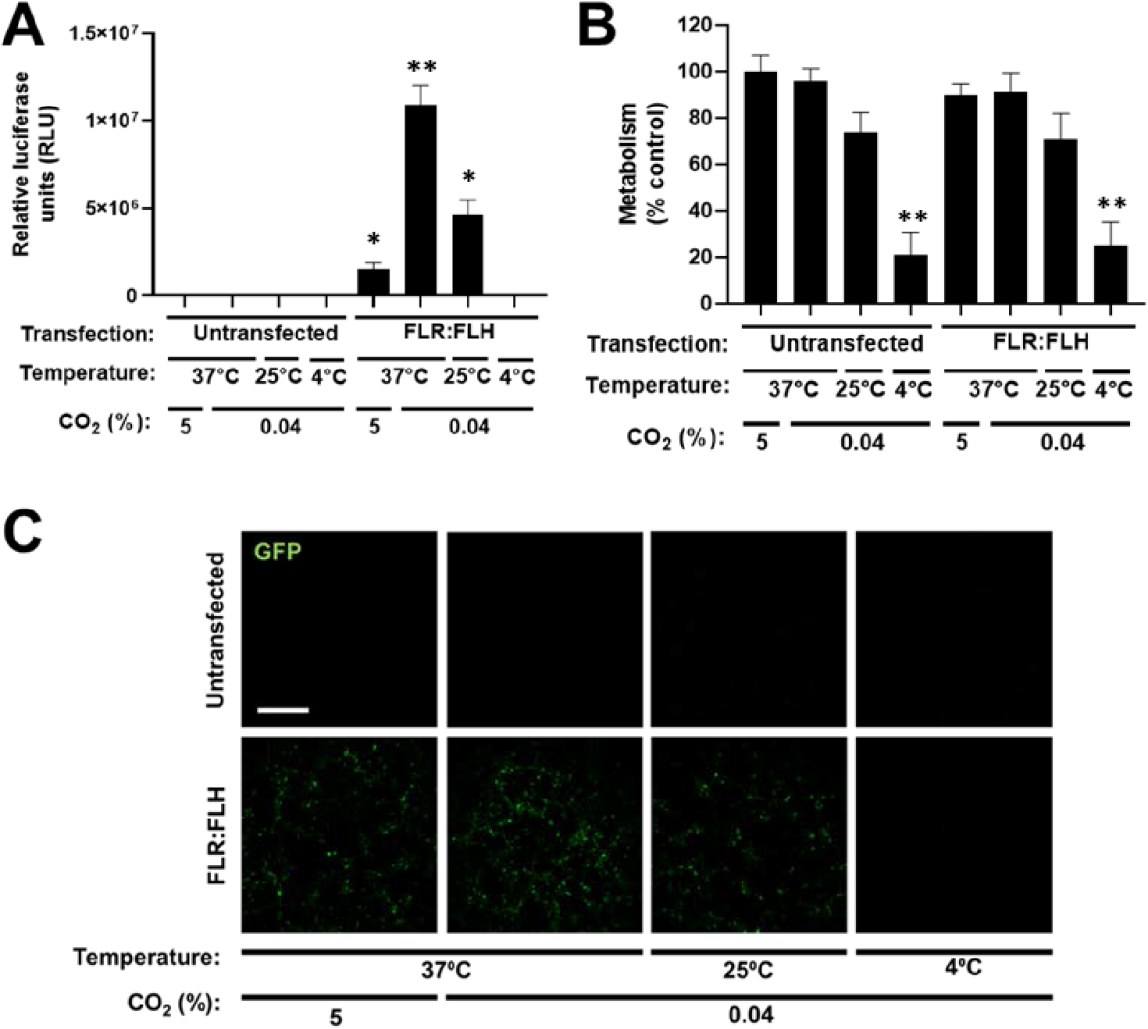
Effect of temperature and CO_2_ saturation on transfection efficiency of FLR:FLH in NIH3t3 cells. (A) Luciferase assay of pGluc pDNA transfection at different temperatures and CO_2_ supplementation using FLR:FLH at 24 h using 1× dose. (B) Metabolic activity (PrestoBlue) of NIH3t3 cell monolayers transfected as in (A). Data were normalized to untransfected (Untr) as 100% (*N* = 6, bars denote SD; **p < 0.01, *p < 0.05). (C) Fluorescence microscopy of pGFP DNA transfection as for (A) (bar is 250 μm).

Extending these analyses to 4 °C incubation (atmospheric gas/pressure in a refrigerator, Figure S2) there was a clear decrease in metabolism (19.2% of control; Figure S2A) and an increase in dead cells (33%) (Figure S2B). We therefore concluded that room-temperature and atmospheric CO_2_ conditions were indeed compatible with short-term (24 h) incubation of cells. We next assessed transfection under these conditions (Figure 2). Interestingly, transfections conducted at atmospheric CO_2_ had significantly more effective transfection (with no effect on viability themselves, Figure 2A,B) over those in conventional culture conditions (∼6 and ∼4-fold at 37 and 25 °C, respectively) with negligible transfection at 4 °C. We repeated experiments using a cell-autonomous fluorescent reporter enhanced-green fluorescent protein (enhanced GFP) pDNA (pGFP) to measure transfected cell percentage and transgene expression level with microscopy (Figure 2C). These data confirmed a trend similar to that of the Gluc reporter.

It was obvious from media color change that atmospheric samples were at a much higher alkaline pH than those supplemented by CO_2_ due to the nature of conventional media buffering; those at atmospheric conditions achieving over pH 8.1 when tested. We therefore investigated the effect of altering the pH of transfection media (growth media: GM) by addition of NaOH. This was conducted in conventional CO_2_ incubators and atmospheric conditions at 37 °C (Figure S3). We also tested the effect of more tightly controlling pH with CO_2_-independent media, CIM. This media does not respond to CO_2_ levels when buffering cultures and therefore was not responsive to culturing in conventional CO_2_ incubators (Figure S4). GM (pH 7.6) was tested compared with that up to pH 9.58. Transfections in 5% CO_2_ showed a dramatic increase in transfection efficiency with increasing alkalinity (Figure S3) without effect on metabolism; however, it was clear that the highest pH samples tested had been buffered toward neutrality (e.g., pH was 9.58 at the onset and after incubation was ∼pH 8.1). When repeated at atmospheric CO_2_ without pH buffering, the highest alkalinity samples were not cell viable and yielded no transfection; however, the trend was maintained with higher pH generating more effective transfection up to pH 8.35 (Figure S3).

To remove the responsive buffering system, we employed CIM that contains a unique buffering system composed of mono- and dibasic sodium phosphate and β-glycerophosphate, supplemented with fetal bovine serum (FBS) similar to that of GM (Figure S4). CIM is formulated with components that enhance cellular production and utilization of CO_2_ such that an exogenous source is not required for maintenance of CO_2_-dependent cellular functions, and therefore can be directly compared to conventional CO_2_-incubator culture. CIM was compatible with cell transfection (Figure S4A) and metabolism (Figure S4B) but as it maintained its pH 7.6 during atmospheric culturing with cells, it did not enhance transfection by change in pH. Adjusting CIM pH, which is stable in atmospheric conditions, confirmed that ∼pH 8.0–8.3 appeared the most optimal for transfection using GET nanoparticles. GFP-transfection data mirrored that of Gluc, with increased transfection and brighter transfected cells with alkaline pH, respectively (Figures S3 and S4).

In conclusion, temperature was a significant variable (20–37 °C suitable for experiments, but not 4 °C), whereas CO_2_ levels for these short (24 h) experiments had no effect on viability or transfection efficiency when corrected for pH (Figures S2 and S4). Alkaline pH during incubation was transformative for transfection. Importantly, this was not a direct effect on *gaussia luciferase* reporter protein activity itself in the control experiments (Figure S5).

### 3.3 Serial Delivery to Retain and Augment High Transgene Expression

**Figure 3:**
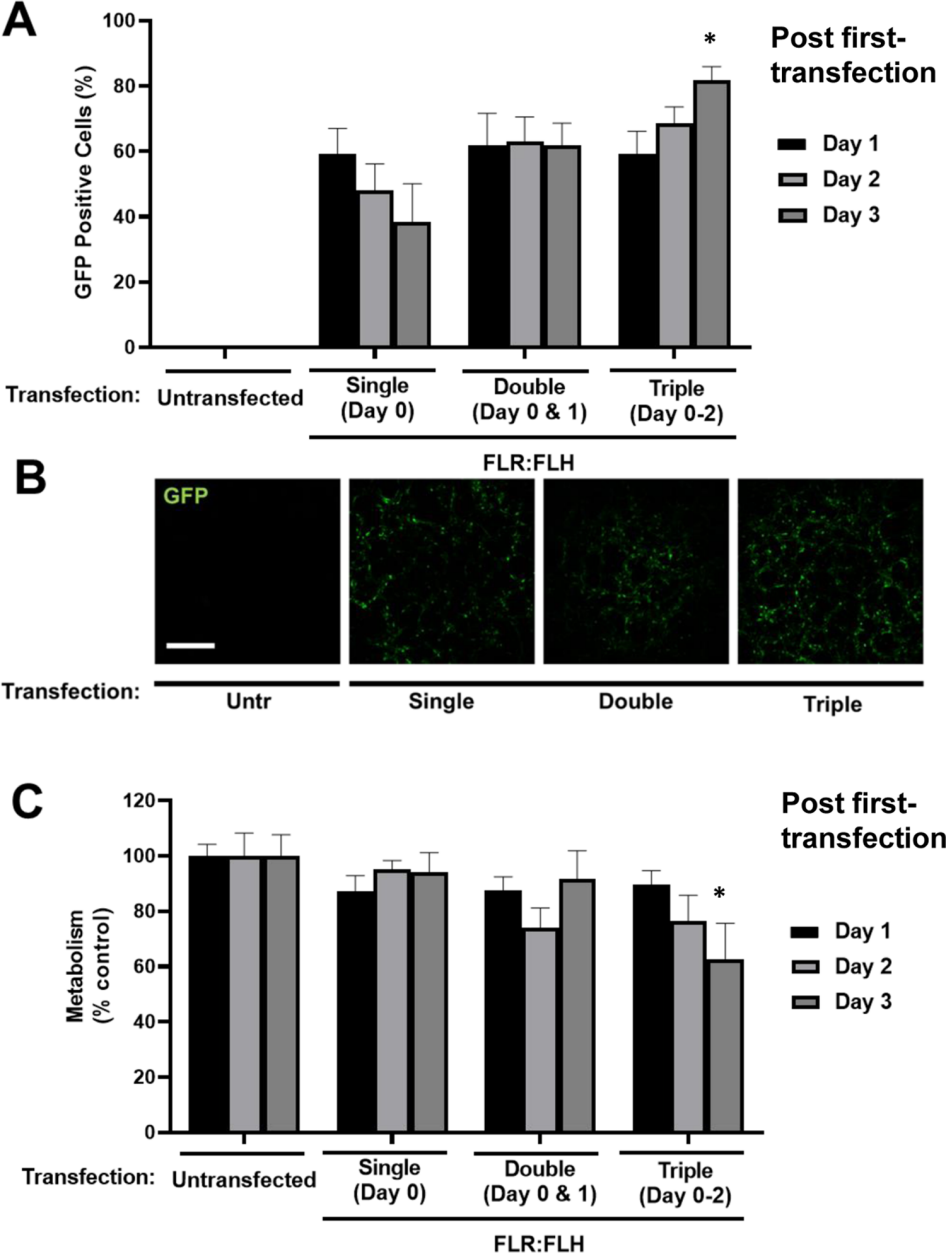
Effect on transfection efficiency and metabolic activity of FLR:FLH serial delivery in monolayers of NIH3t3 cells. (A) Flow cytometry quantification of pGFP DNA transfection using FLR:FLH 1× dose delivered as single (day 0), double (days 0 and 1), or triple (days 0–2) dosages per day. (B) Fluorescent imaging of GFP transfection at day 3 (bar is 250 μm). (C) Metabolic activity (PrestoBlue) of NIH3t3 cell monolayers transfected in (A) (*N* = 6, bars denote SD; **p < 0.01, *p < 0.05).

Given that transfection with moderate doses (1× and below, FLR: FLH) was effective at transfecting cells without significant effect on viability or metabolism, we next tested if daily delivery could retain and augment gene expression in transfected cells (Figure 3). We used pGFP and were able to show that further dosages of transfection were able to build percentage GFP positive levels at day 3 (38.4 ± 11.6% with single transfection) versus successive daily dosing (61.8 ± 6.8% with two, 81.7 ± 4.2% with three doses) (Figure 3A). Furthermore, the highest levels of expression over the three-day period (days 1–3) were retained with serial dosing meaning reduction in percentage transfected was prevented and increased over the period with daily transfections. With serial delivery, there was an increasing negative effect on metabolism. Interestingly, metabolism recovered overtime to untransfected levels showing that it was possible to build and maintain expression in cells with multiple dosing, which was cell viability compatible (Figure 3B,C).

### 3.4 Applying Positive (PP) and Negative (NP) to Cell Culture

**Figure 4:**
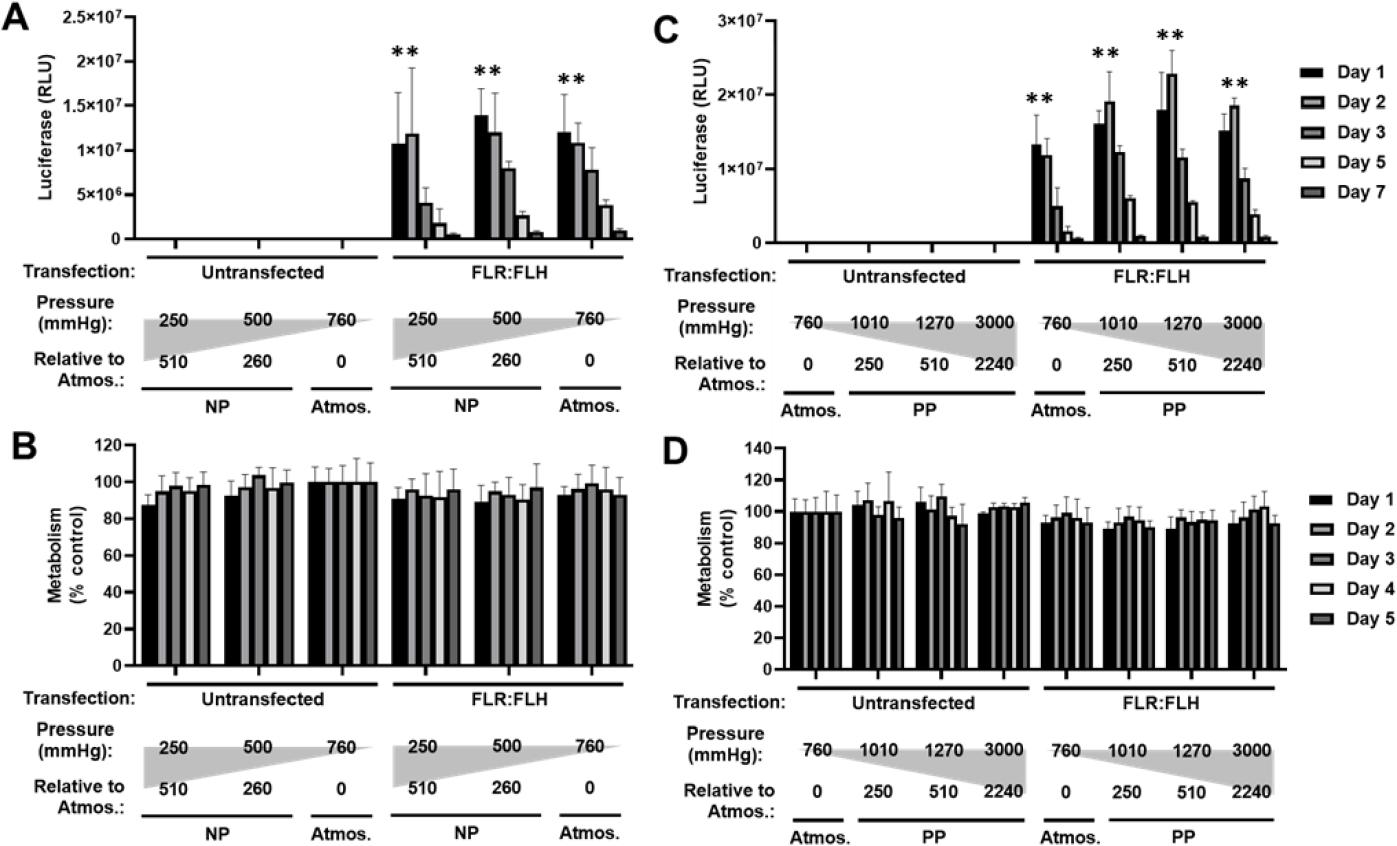
Effect of negative (NP) and positive (PP) pressures on transfection efficiency of FLR:FLH in monolayers of NIH3t3 cells. Luciferase assay of pGluc pDNA transfection using FLR:FLH 1× dose exposed to (A) negative (NP) or (C) positive (PP) pressures during the experiment. Metabolic activity (PrestoBlue) of NIH3t3 cell monolayers exposed to (B) negative (NP) or (D) positive (PP) pressures during the experiment. Data were normalized to atmospheric pressure (760 mmHg) as 100% (*N* = 6, bars denote SD; **p < 0.01, *p < 0.05).

We devised systems that could apply positive (PP) and negative (NP) pressures (compared to atmospheric) experimentally by using a humidified compressed-air pressure vessel or vacuum oven, respectively. We determined that evaporation was not an issue when humidified and could stably retain the 37 °C (body temperature) or room-temperature (20–25 °C) within the systems. However, we could not achieve conventional 5% CO_2_ culture conditions, so for cell experiments, we employed CIM media to control for variation in pH, which would affect transfection efficiency and viability. We tested NP to PP (+510 to +2240 mmHg) exposing cell monolayers for 24 h (Figure 4). There was no immediate or long-term effect (following 4 days) on metabolism compared to control cells (Figure 4B,D).

### 3.5 The Effect of Negative Atmospheric Pressure on Transfection of Cells

**Figure 5:**
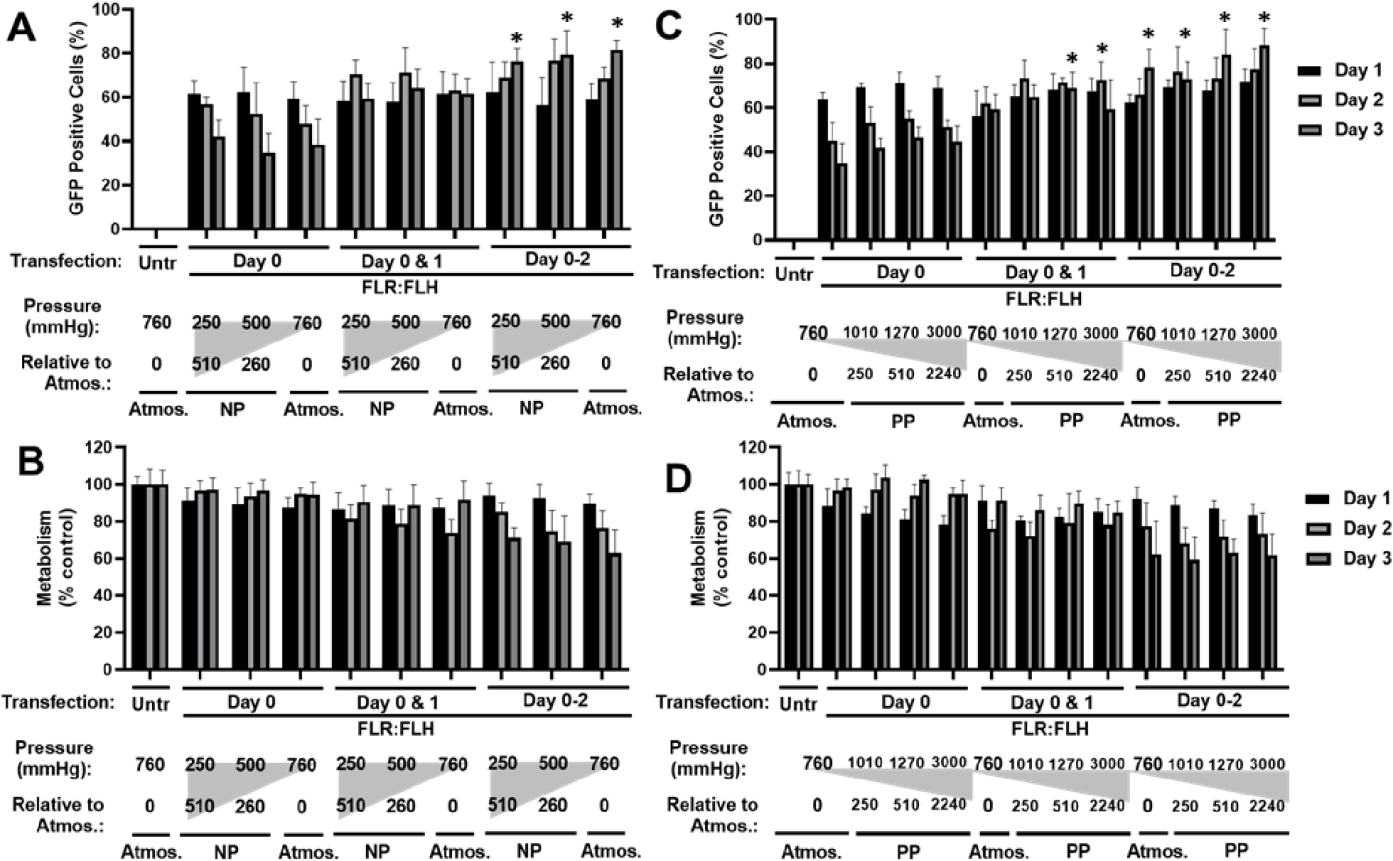
Effect of negative (NP) and positive (PP) pressures on transfection efficiency of FLR:FLH serial delivery in monolayers of NIH3t3 cells. Negative pressure (NP): (A) flow cytometry of GFP pDNA transfection using FLR: FLH 1× dose delivered once (day 0), twice (day 0 and 1), or thrice (days 0–2) exposed to negative (NP) pressure. (B) Metabolic activity (PrestoBlue) of NIH3t3 cell monolayers exposed to negative (NP) pressure. Positive pressure (PP): (C) and (D) (as for a and B). Data were normalized to untransfected cells at atmospheric pressure (760 mmHg) as 100%. (*N* = 6, bars denote SD. Statistical tests were performed between atmospheric and test samples, all comparisons were not significant except for those shown as *p < 0.05.)

We next assessed transfection efficiency and persistence in cell monolayers under NP. NP from atmospheric pressure (760 mmHg) to 250 mmHg (−510 mmHg down) showed little changes in transfection efficiency (1.21 × 10^7^ ± 0.42 versus 1.08 × 10^7^ ± 0.65 RLU, respectively) with pGluc DNA (Figure 4A). We then repeated transfections with pGFP DNA, which correlated to luciferase transfections with 68.6 ± 4.9% and 63.8 ± 3.8% positivity, respectively (Figure 5A). Further to these, we conducted serial transfections (where cells were removed from the chamber/vacuum to administer the transfection daily) (Figure 5), which could achieve 81.7 ± 4.2% and 76.4 ± 6.0% GFP positivity (Figure 5A). Initial metabolic activity dropped to 85.1 ± 6.5%, but it was clear from single and double dosing that cells were able to fully recover postfinal application of transfection (Figure 5B). These data lead to the conclusion that atmospheric NP per se does not enhance transfection but is compatible with transfection by GET nanoparticles.

### 3.6 The Effect of Positive Atmospheric Pressure on Transfection of Cells

Next, we assessed and optimized reporter gene transfer (transfection) efficiency and persistence in cell monolayers under PP. Using single transfection of pGluc at atmospheric pressure (760 mmHg) to a PP of 3000 mmHg (+2240 mmHg increase), we observed a small but significant enhancement in transfection efficiency (1.34 × 10^7^ ± 0.55 versus 1.61 × 10^7^ ± 0.28 RLU for atmospheric and 1010 mmHg, respectively) (Figure 4). GFP transfection correlated with this (63.8 ± 3.1% and 69.1 ± 1.8% positivity for atmospheric versus 1010 mmHg PP, respectively) (Figure 5C). We repeated daily (serial) transfection, which could achieve higher positivity (84.0 ± 8.4% and 88.3 ± 7.6%) with initial metabolic activity dropping (78.4 ± 4.8%) acutely (not significant statistically) but almost fully recovering (95.1 ± 3.3% at 2 days post-transfection) (Figure 5D).

**Figure 6:**
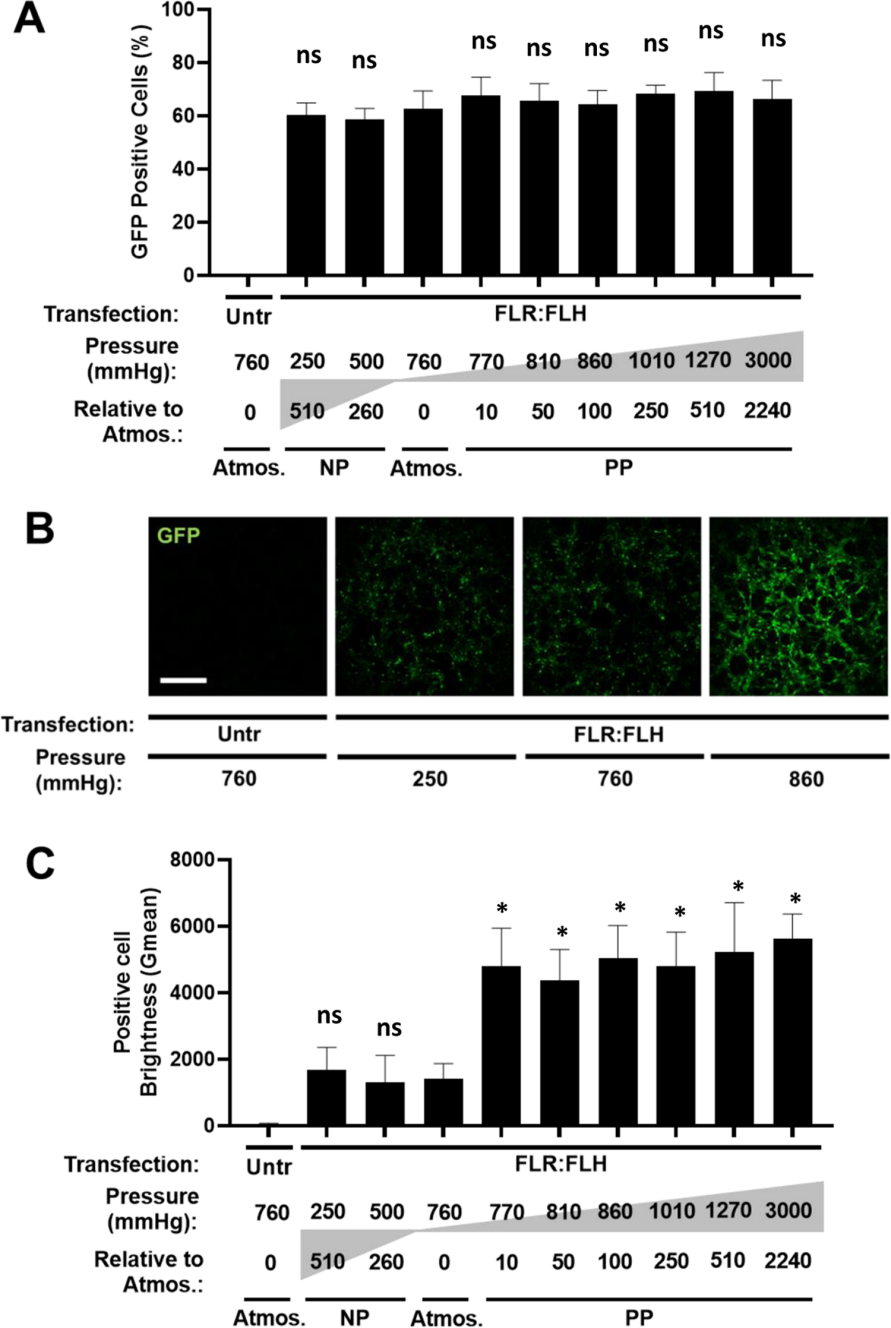
Effect of pressure on transfection efficiency and expression level of FLR:FLH in monolayers of NIH3t3 cells. (A) GFP positive cells (determined by flow cytometry) of pGFP pDNA transfection using FLR:FLH 1× dose exposed to different pressures during transfection (*N* = 6, bars denote SD). (B) Fluorescence microscopy of pGFP pDNA transfection as for (A) (bar is 250 μm). (C) Metabolic activity (PrestoBlue) of NIH3t3 cell monolayers transfected as in (A). Data were normalized to untransfected (Untr) as 100%. (*N* = 6, bars denoteSD, statistical tests were performed between atmospheric and test samples, ns is not significant, *p < 0.05).

Unlike NP administration, it was clear from flow data (Figure 6A) and microscopy (Figure 6B) that cells treated with PP were brighter for GFP signal (>3- to 5-fold *G*_mean_ than atmospheric controls) (Figure 6C). The improvement in efficiency of transfection brightness with PP was saturated at relatively low increase in PP (+10 mmHg over atmospheric pressure), with the highest PP not benefiting further (no significant difference 770–3000 mmHg) (Figure 6C). These data demonstrate that atmospheric, NP, or PP administration is compatible with effective GET transfection and that serial transfection is useful in increasing transfected cell levels (number of cells and level of expression).

## 4 Discussion

### 4.1 Low Dose Effective Transfection

Viruses, such as lentivirus, are usually employed at the multiplicity of infection (MOI) of one virus/cell (1 MOI) or for difficult-to-infect cells up to 100 viruses/cell (100 MOI) in cultured cells. We have determined that for our most optimal formulation, FLR:FLH, the lowest dose to exhibit significant transfection over background was 0.02x (2.5 ng dose), which proportionally increased to plateau at the 2× (0.25 μg) dose. To compare with viral strategies, for 0.5 × 10^5^ cells (confluent well), a 1× dose (0.125 μg) is ∼2.3 × 10^10^ plasmids (5 kb size), and represents a transfection of ∼5 × 10^5^ plasmids/cell. At the lowest tested dose 0.02X (2.5 ng), this correlates to 1 × 10^4^ plasmid/cell or a multiplicity of transfection of 10,000, i.e., 100 times that of the highest generally employed with viruses. This difference in effective copies/cell to generate gene expression in treated cells shows the ineffectiveness of nonvirus gene delivery. However, it must be noted that we tested pDNA delivery, which is much less effective to transfect than mRNA, which requires only cytoplasmic, not nuclear localization for expression. Furthermore, viruses have evolved over millions of years to infect cells effectively, such that a simplistic complexation of pDNA and a synthetic peptide, getting closer to viral dose levels in this study, is encouraging. The data from our previous work show that cell association and uptake is not the bottleneck in nonviral gene delivery but endosomal escape and trafficking to the nucleus in the optimal transcription-competent format is lacking. It is clear that only a small proportion of 10,000 copies make it to the desired nuclear localization in a form that is transcriptionally functional, so further efforts to improve efficacy as we move toward translation are still required to fully exploit nonviral nanotechnologies.

The difference between low dose transfection using FLR alone or that supplemented with endosomally escaping variants is profound. Although low, transfection significantly above the background was detected in the FLR:FLH formulation at the lowest dose tested, 50-fold lower than a conventional transfection. It is well established that a threshold level of uptake is required for detectable levels of transgene expression, and that uptake kinetics can dictate the efficacy of successful endosomal escape and nuclear localization of DNA in transfections. Endosomal escape potential can be scrutinized via investigating peptides buffering capacity,^30^ hemolytic activity,^31^ and/or tracking endosomes using staining assays to confirm nuclear localization.^32^ We hypothesize that even at low doses, FLH-containing formulations present to be more effective in this bottleneck, meaning that lower DNA levels can yield transfection success.

### 4.2 Alkaline pH and Physiological Temperature is Beneficial for the Highest Transfection Efficacy

Nanoformulations, such as GET nanoparticles, are highly affected by salt concentration and pH in relation to their size and charge. We have shown that formulations generated in a neutral serum-free environment can transfect cells effectively and on exposure to cells in cytocompatible alkaline media conditions (<pH 8.3) the transfection efficacy is significantly enhanced. The mechanism for this enhancement is unclear; however, it may be linked to endosomal buffering (and increase ionization/positive charge of the GET peptides, p*K*_a_) and escape, which is highly affected by internal vesicle pH. They act as proton sponges with subsequent flux entry of chloride ions into the endosome. This generates osmotic pressure that eventually leads to rupture and endosomal escape into the cytosol.^33^

### 4.3 Positive Pressure Can Enhance Level and Longevity of Transgene Expression

An interesting observation from controlling atmospheric pressure during transfection was that a small but significant increase in atmospheric pressure could yield more significant transgene expression in individual cells (assessed by GFP expression and flow cytometry); this also appears to prolong expression (Figure 5C). Pressure-mediated augmented transfection efficiencies can be often attributed to enhanced nuclear localization,^34^ or cell uptake and permeation.^35^ However, this phenomenon also requires further exploration, focusing on the level of endosomal escape, and experiments to dissect the point at which pressure could play a role, distinguishing between uptake, escape, nuclear localization, vector unpacking, and transcriptional output.

### 4.4 Serial Delivery is Possible with GET to Maintain High Transgene Levels and Transfected Cell Numbers

We have shown that transfecting low doses can yield detectable transgene levels with a sensitive reporter (such as Gluc); however, due to the nontoxic nature of GET and a wide window of efficacy with negligible viability or proliferation effects after an initial inhibition of metabolism, we were able to demonstrate daily transfection regimens (Figure 5). Daily transfection with nontoxic doses could offer low effects on metabolism/viability that were transient and allow successive building of transgene expression level in cultures (Figure 5, three transfections, days 0–2). More conventional transfection reagents, such as Lipofectamine, cannot be used in such a way,^22^ as they have much more significant negative effects on culture proliferation and serial transfections yield nonviable cultures. With GET, this could be a future strategy to reach the higher expression generated using viral systems and should be explored further with chronic dosing strategies.

## 5 Conclusion

Nonviral gene delivery, especially of pDNA, is not as effective as viral-based systems; however, there are several benefits including cost, bioprocessing, stability, and immunogenicity using nanotechnological approaches to transfection and gene therapy. It was important to understand the microenvironmental parameters that can affect nonviral gene delivery, and we have confirmed the requirement for physiological temperatures and the benefit of alkaline pH and positive pressure in improving transfection efficacy for our GET system. Employing nonviral gene delivery in a tractable format to aid regenerative medicine approaches, including gene therapies, could have immense impact for several disorders. Having the optimal conditions for gene transfer will facilitate new drug delivery strategies and allow for approaches to deliver the activity of novel nanotechnologies and gene therapeutics.

## Supporting Information Available

Figure S1: Effect of temperature and CO_2_ saturation on NIH3t3 cell viability; Figure S2: Effect of low temperature and CO_2_ saturation on NIH3t3 cell viability; Figure S3: Effect of pH and CO_2_ saturation on transfection efficiency of FLR:FLH in NIH3t3 cells; Figure S4: Effect of media buffering and CO_2_ saturation on NIH3t3 cell viability; Figure S5: Direct effect of media pH on luciferase enzyme activity (PDF)
