# Supplementary material for: Effects of Microenvironment and Dosing on Efficiency of Enhanced Cell Penetrating Peptide Nonviral Gene Delivery
Source: ACS Omega. 2024 Jan 18;9(4):5014–23. doi: 10.1021/acsomega.3c09306 (PMC10831962; doi:10.1021/acsomega.3c09306)
Supplement: Supplementary file 1 — ao3c09306_si_001.pdf [file ao3c09306_si_001.pdf]

Supplemental Information:

Effects of microenvironment and dosing on efficiency of enhanced cell penetrating peptide non-viral gene delivery

James E. Dixon[1,2*], Vanessa Wellington[1], Alaa Elnima[1] and Hoda M. Eltaher[1]

[1]Regenerative Medicine & Cellular Therapies Division, The University of Nottingham Biodiscovery Institute (BDI), School of Pharmacy, University of Nottingham, Nottingham, NG7 2RD, UK. [2]NIHR Nottingham Biomedical Research Centre, University of Nottingham, Nottingham, UK

*Correspondence should be addressed to James E. Dixon:

james.dixon@nottingham.ac.uk

Regenerative Medicine & Cellular Therapies,

The University of Nottingham Biodiscovery Institute (BDI),

School of Pharmacy

University Park

University of Nottingham

Nottingham NG7 2RD, UK

Tel: +44 (0) 115 7486313

Keywords. GAG-binding enhanced transduction (GET); Temperature, CO_2, Serial delivery, Pressure, Gene transfer, transfection.

Figure S1. Effect of temperature and CO_2 saturation on NIH3t3 cell viability.

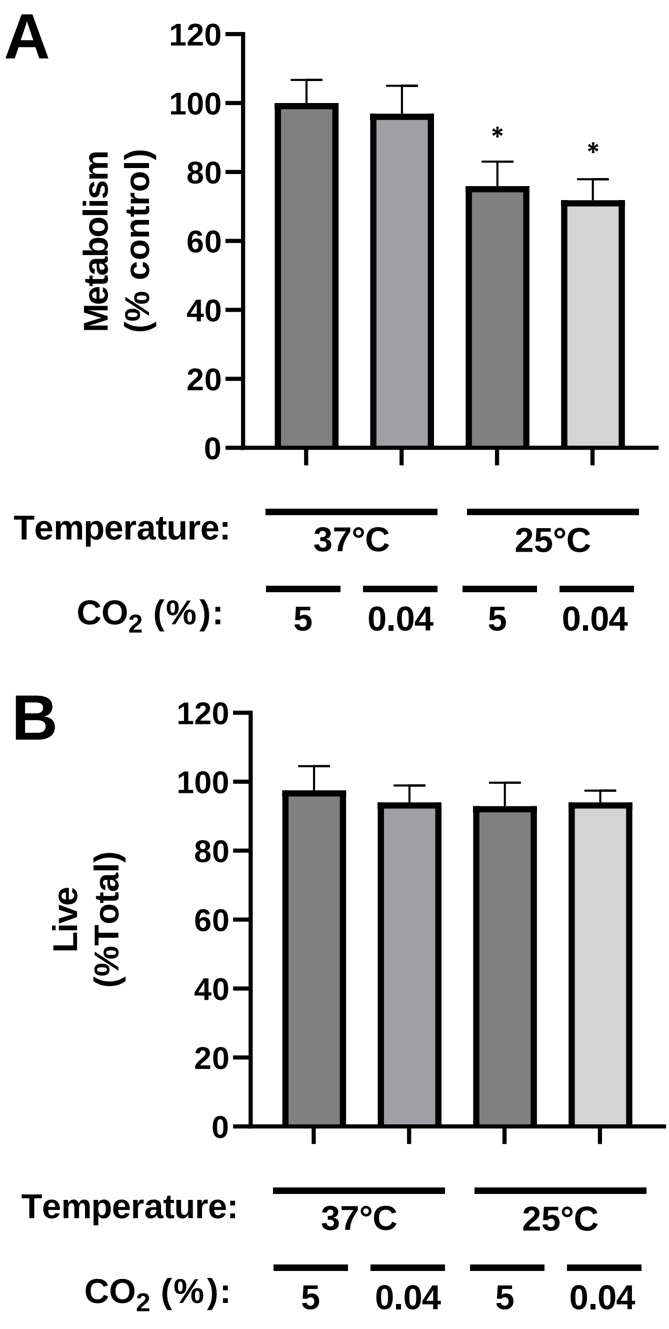

Figure S1. Effect of temperature and CO_2 saturation on NIH3t3 cell viability. A) Metabolic activity (Alamar Blue) of NIH3t3 cell monolayers incubated at room temperature (25'C) and at 37'C in atmospheric conditions (0.04%) or supplemented with CO_2 (5%) for 24h. Data was normalized to 37'C, 5% CO_2 as 100%. B) Live/dead staining quantification using the conditions from A) (N=6, bars are S.D. * $p<0.05$).

Figure S2. Effect of low temperature and CO_2 saturation on NIH3t3 cell viability.

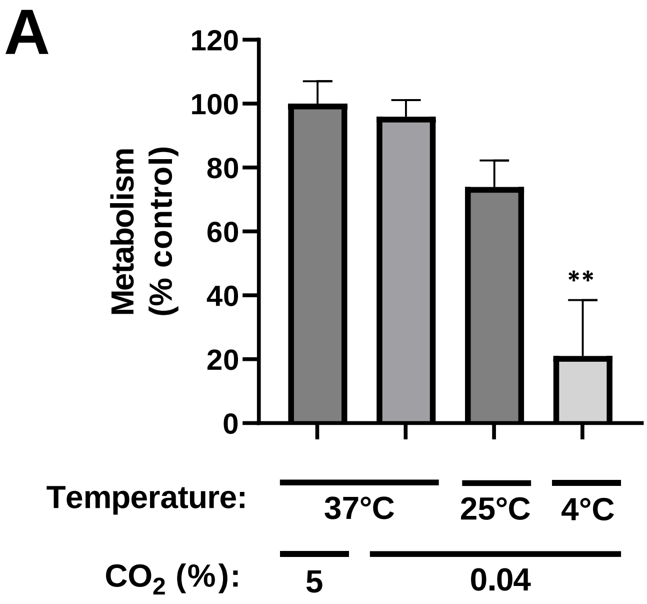

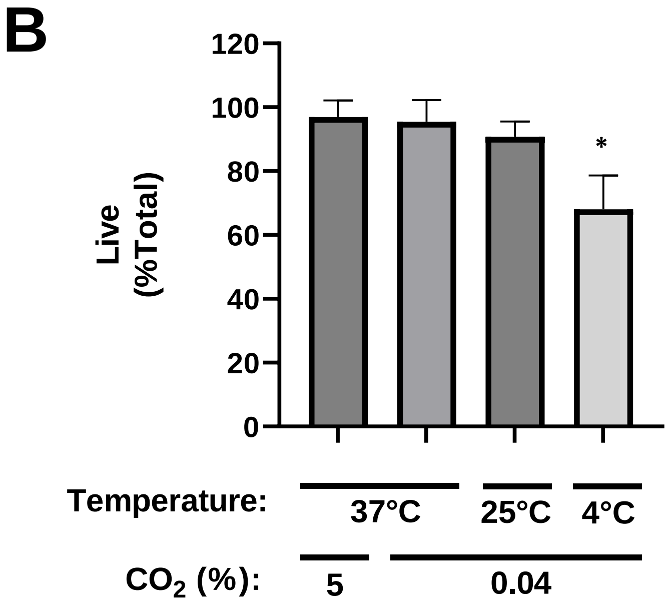

Figure S2. Effect of low temperature and CO_2 saturation on NIH3t3 cell viability. A) Metabolic activity (Alamar Blue) of NIH3t3 cell monolayers incubated at 4'C, room temperature (25'C) and at 37'C in atmospheric conditions (0.04%) or supplemented with CO_2 (5%) for 24h. Data was normalized to 37'C, 5% CO_2 as 100%. B) Live/dead staining quantification using the conditions from A) (N=6, bars are S.D. ** $p<0.01$, * $p<0.05$).

Figure S3. Effect of pH and CO_2 saturation on transfection efficiency of FLR:FLH in NIH3t3 cells

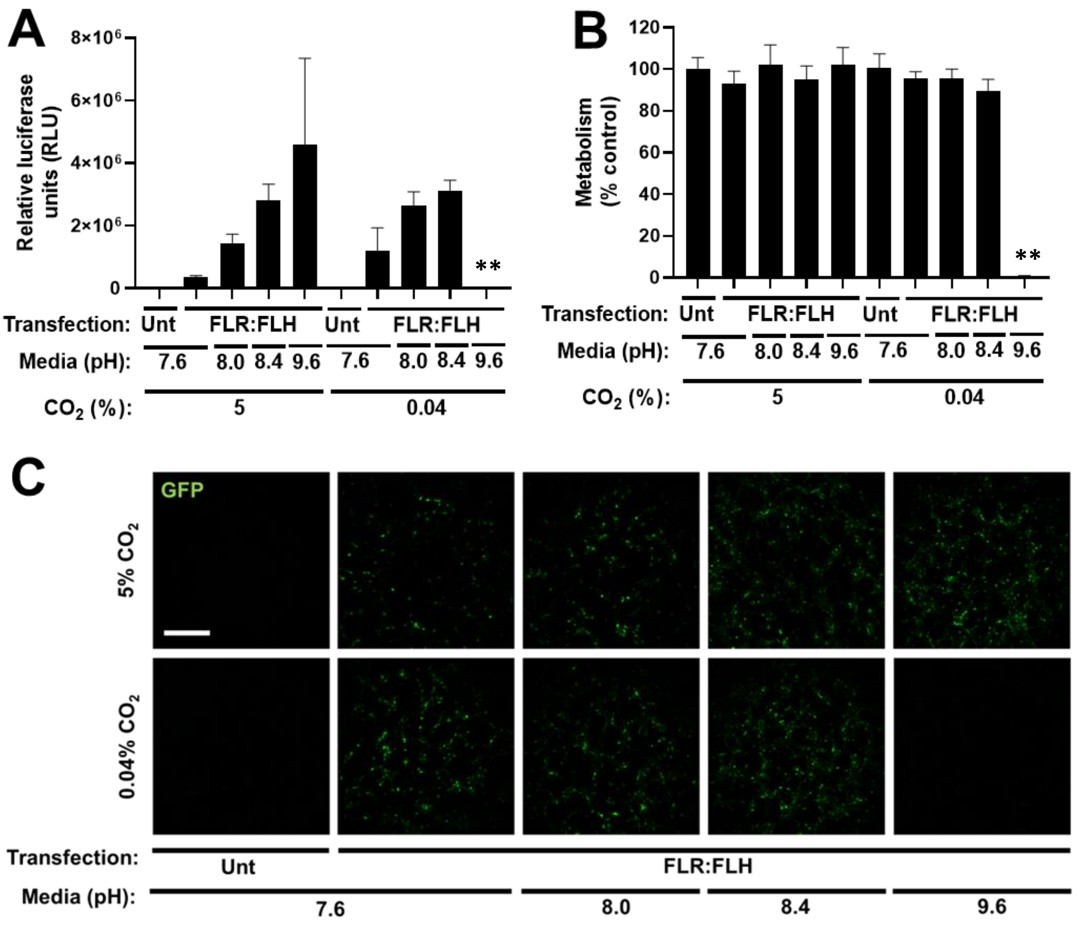

Figure S3. Effect of pH and CO_2 saturation on transfection efficiency of FLR:FLH in NIH3t3 cells. A) Luciferase assay of pGluc pDNA transfection at different pHs and CO_2 supplementation using FLR:FLH at 24h using 1X dose. B) Metabolic activity (PrestoBlue) of NIH3t3 cell monolayers transfected as in A). Data was normalized to untransfected (Untr) as 100%. (N=6, bars are S.D. ** $p<0.01$). C) Fluorescence microscopy of pGFP pDNA transfection as for A) (bar is 250µm).

Figure S4. Effect of media buffering and CO_2 saturation on NIH3t3 cell viability.

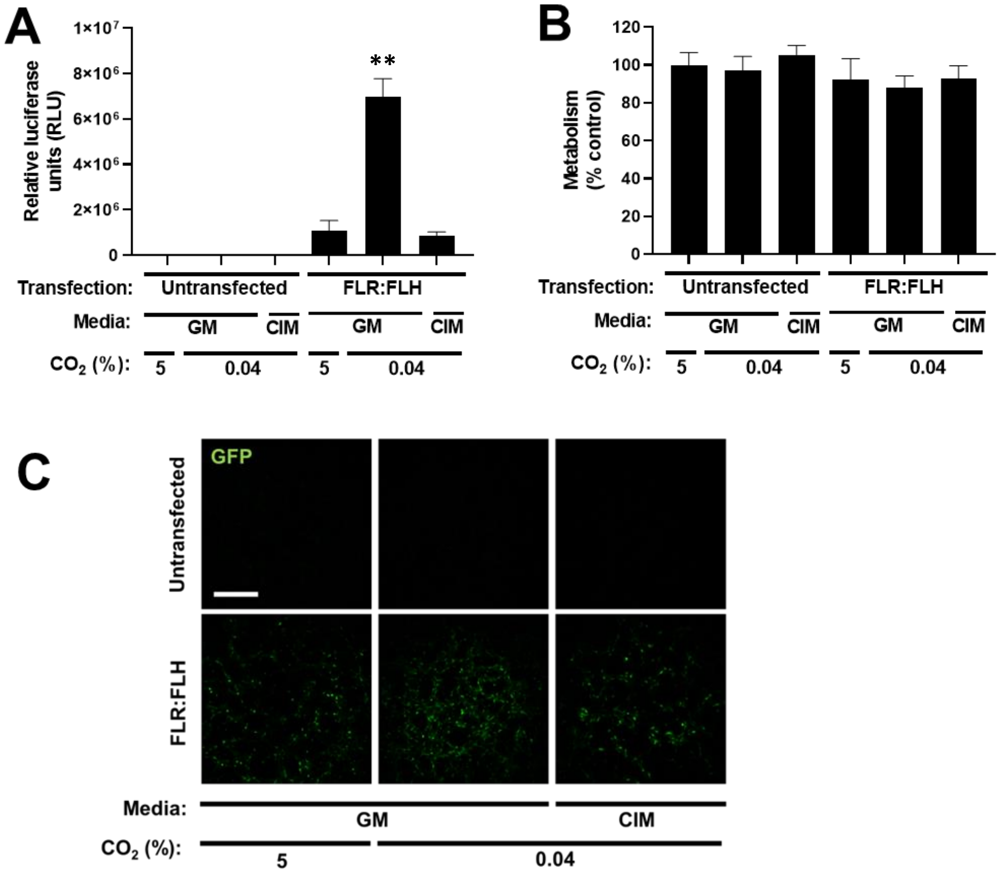

Figure S4. Effect of media buffering and CO_2 saturation on transfection efficiency of FLR:FLH in NIH3t3 cells. A) Luciferase assay of pGluc pDNA transfection in growth media (GM) or CO_2-independent media (CIM) and CO_2 supplementation using FLR:FLH at 24h using 1X dose. B) Metabolic activity (PrestoBlue) of NIH3t3 cell monolayers transfected as in A). Data was normalized to untransfected (Untr) as 100%. (N=6, bars are S.D. ** p<0.01). C) Fluorescence microscopy of pGFP pDNA transfection as for A) (bar is 250µm).

Figure S5. Direct effect of media pH on Luciferase enzyme activity

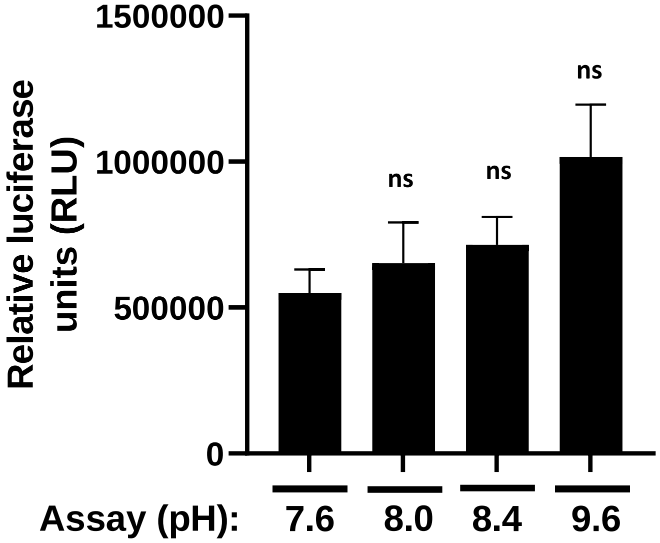

Figure S5. Direct effect of media pH on Luciferase enzyme activity. Media containing expressed Gaussia luciferase collected from a single transfection diluted in different pH buffers was assayed to assess the effect of pH on enzyme activity (N=6, bars are S.D. ns is not significant).
